# Psychometric Properties of the Lasher and Faulkender Anxiety about Aging Scale (AAS) among Iranian Older Adults

**DOI:** 10.3390/ejihpe11030060

**Published:** 2021-07-28

**Authors:** Amir H. Pakpour, Shamsedin Namjoo, Khadijeh Sabahiazar, Mohammad Asghari Jafarabadi, Vijay Kumar Chattu, Hamid Allahverdipour

**Affiliations:** 1 Social Determinants of Health Research Center, Research Institute for Prevention of Non-Communicable Diseases, Qazvin University of Medical Sciences, Qazvin 3419759811, Iran; apakpour@qums.ac.ir; 2 Department of Nursing, School of Health and Welfare, Jönköping University, 553 33 Jönköping, Sweden; 3 Department of Aging, University of Social Welfare and Rehabilitation Sciences, Tehran 1985713834, Iran; shamsadinnamjoo@yahoo.com; 4 Department of Health Education & Promotion, Tabriz University of Medical Sciences, Tabriz 14711, Iran; s.sabahi1357@gmail.com; 5 Road Traffic Injury Research Center, Department of Statistics and Epidemiology, Tabriz University of Medical Sciences, Tabriz 14711, Iran; asgharimo@tbzmed.ac.ir; 6 Division of Occupational Medicine, Department of Medicine, Temerty Faculty of Medicine, University of Toronto, Toronto, ON M5C 2CS, Canada; 7 Department of Public Health, Saveetha Medical College, Saveetha Institute of Medical and Technical Sciences, Saveetha University, Chennai 600077, India; 8 Research Center of Psychiatry and Behavioral Sciences, Tabriz University of Medical Sciences, Tabriz 14711, Iran

**Keywords:** aging, anxiety, cross-sectional design, depression, Iran, lasher anxiety aging scale, psychometric, old age

## Abstract

(1) Background: The older adult population of society is exposed to multiple stressors daily, such as the loss of loved ones, dysfunctional mobility, financial dependence, and suffering from numerous chronic illnesses. The present study aimed to assess the psychometric properties of the Anxiety about Aging Scale among older adults in Iran. (2) Methods: A sample of 703 community-dwelling older adults was recruited and screened using a standardized tool. The mean age of participants was 69.4 ± 8.1 years. The majority of participants were male (59.2%), married (66.6%), and illiterate (79.7%). A ‘forward-backward’ translation method was used in developing the Iranian version of the AAS for assessing the psychometric properties among older adults. Confirmatory factor analysis (CFA) and the Rasch model were used for construct validity. (3) Results: Applying CFA indicated that the model’s four original factors are the best solution, representing 55% of the total variance. The result of the CFA showed that this four-factor model had a good fit for the data. The findings were also confirmed by Rasch analysis. (4) Conclusions: The Persian version of the AAS is valid and reliable for measuring aging anxiety among Iranian older adults.

## 1. Introduction

Increases in life expectancy, the development of medical facilities, and improvements in welfare activities have all contributed to an increase in the elderly population worldwide [1]. Today, older people have a healthier life and can work more than they did in the past [2]. On the other hand, today, many people have a negative attitude regarding aging and experience anxiety related to aging due to loss of functional independence, dependence on others, and increases in chronic illness [3]. In contrast, only a small percentage of older people suffer from these problems, and most of them (more than 80%) have the necessary functional independence in their lives [4].

As people get older, their attitudes toward old age and their attitudes toward older people become critical in terms of age group relationships. People’s perceptions of their aging are also likely to be influenced by their age group identity processes. In fact, aging anxiety has been shown to be a variable associated with increased levels of negative attitudes toward older people [5]. Moreover, the negative perception of aging leads to enhanced anxiety levels, which is termed anxiety of aging [6]. It is also postulated that aging anxiety refers to worrying about the capacity to maintain health in such dimensions as the psychological, social, physical, and transpersonal or spiritual realms [7]. As a result, understanding how aging anxiety can influence behavior and interactions is a critical issue. The anxiety of aging denotes anxiety about growing old, which is likely influenced by culture, gender, age, and socioeconomic conditions [8]. Studies have also demonstrated that people have varied perceptions of aging anxiety levels, such as low or very high anxiety levels. In general, individuals suffering from anxiety in age-old have a reduced tendency to socialize with other older adults [9].

Additionally, some studies have reported that aging anxiety is more likely to be reflected among middle-aged groups [10]. Psychological issues related to the anxiety of aging might lead to numerous consequences such as depression, cognitive impairment [11], lower life satisfaction [12]. Studies have shown that the tendency to exhibit a decline in health status is higher among people who suffer from the anxiety of aging [8]. On the other hand, anxiety in old age leads to an increase in the incidence of chronic diseases, such as diabetes mellitus, as well as mental disorders that can create high costs in terms of care for individuals, families, communities in addition to creating a huge burden on health systems [8]. Several factors can reduce the anxiety of aging and ultimately improve quality of life. Studies have shown that the experiences of the elderly during their lifetime have an adverse effect on anxiety in old age [10,13]. Hence, the more experiences people have can be a protective effect for the person against anxiety caused by old age. Self-efficacy is one of the most important factors that can be used to achieve healthy and successful aging and reduce aging anxiety. Self-efficacy was identified as an important variable to successfully adapt to various changes in new transitional periods of people’s lives [10].

Aging anxiety is a critical issue and can be influenced by all aspects of the older adult’s life; additionally, perceptions of aging as well as anxiety of aging are different in various cultures, economic and social situations around the world [12]. Therefore, designing appropriate interventions is necessary to reduce aging anxiety and improve quality of life. Therefore, valid and reliable tools are required to investigate the anxiety of aging and potential factors escalating it, especially among the aging population. Most of the scales evaluating aging anxiety are unidimensional, and the item factor loadings are generally small [14]. However, concrete diagnoses of the anxiety of aging require several components, and as a result, multi-dimensional scales might be a proper tool.

Furthermore, one-dimensional scales suffer from applicability limitations to distinguish various dimensions of anxiety among older adults. One of the common and standard multi-dimensional scales that is used to assess anxiety of aging is Lasher’s Anxiety about Aging Scale [AAS]. This scale was developed by Lasher and Faulkender in 1993 [15]. Since Lasher’s AAS or similar tools are not prepared for psychometrics for people who speak in the Persian language, a psychometric evaluation of these tools is essential for the following reasons: (1) psychometric tools are appropriate for assessing the anxiety of older adults; (2) to investigate the factors that affect aging anxiety among older adults; (3) to investigate the role of gender and age patterns on aging anxiety across the lifespan among the older adults; and (4) to assess the concurrent validity of the AAS. Therefore, with this given background, the present study aimed to assess the psychometric properties of the Anxiety about Aging Scale among older adults in Iran.

## 2. Materials and Methods

### 2.1. Participants

In this cross-sectional design, a sample of 703 community-dwelling older adults were recruited using a multistage random sampling method. The data collection was performed between 1 February and 31 May 2018 in Bukan and Qazvin cities of Iran. The general population covered by the regional health care centers in urban areas formed the sampling frame for this study. In total, 703 older adults participated in this study. The mean age of older adults was 69.4 ± 8.1 years. Most of the participants were male (59.2%), illiterate (79.7%), married (66.6%), and housekeepers by occupation (40.0%). The other characteristics of the participants are shown below (Table 1).

### 2.2. Procedures

In the first stage, out of fifteen urban regions, six regions were selected using proportionate random sampling in two cities. Then, each sample size was calculated using the quota distribution for the older adults registered in health care centers. The inclusion criteria were being at least 60 years old and lack of cognitive disorders (based on physician diagnosis). Additionally, eligibility criteria included (1) willingness to participate in the study and (2) being mentally and cognitively able to be interviewed and complete questionnaires. All prospective participants were informed regarding the study’s objectives, and upon receiving their approval to participate in the study, informed consent was taken from the participants. However, no incentives were provided for participating in this study. The study was conducted according to the guidelines of the Declaration of Helsinki and approved by the Ethics Committee of the Tabriz University of Medical Sciences, Iran [Ethics Reference No: IR.TBZMED.REC.1397.304].

### 2.3. Translation and Adaptation Procedure

The translation procedure was conducted based on international guidelines to confirm the accuracy of the translation. Several steps were involved in this process, which are as follows: (1) forward translation: two independent bilingual native Iranians with expertise in gerontology and geriatrics performed the translation of AAS from English into Persian. (2) Synthesis of the translations: Both translated versions were compared by the research team to combine the translations. (3) Backward translation: two back translations into English were performed by two independent bilingual translators who had not seen the original English questionnaire. These steps were conducted to identify inconsistencies or conceptual errors in the translation procedure. (4) Expert committee: All translators, the study project manager, two health education specialists, and two geriatricians determined the cross-cultural face validity of the AAS. At this stage, some modifications were made to adjust its cultural adaptability and fix any mismatch between the original and translated versions. (5) Piloting: the Iranian (Persian/Farsi) version (Appendix A) of the Lasher Anxiety of Aging Scale (AAS) was pilot tested on ten older adults to understand how they interpret the questionnaire items. The older adults were asked to give their feedback about the clarity and understandability of the scale’s wordings. (6) Field test: all the necessary changes were incorporated based upon the pilot phase recommendations, and the revised version was administered to 703 older adults in Bukan and Qazvin cities.

### 2.4. Measures

Lasher and Faulkender Anxiety about Aging Scale (AAS)

AAS has four subscales that determine the overall aspects of the anxiety of aging. The AAS consists of a 20-item measure (Appendix B) that produces a summative score based on the responses to all 20 items, including: 1. Fear of old people (“I feel very comfortable when I’m around an old person”); 2. Psychological concerns (“I fear it will be tough for me to find contentment in old age”); 3. Physical appearance (“When I look in the mirror, it bothers me to see how my looks have changed with age”); and 4. Fear of losses (“I fear that when I am old, all my friends will be gone”). The Lasher Anxiety of Aging Scale has high internal consistency, and the four factors explain 50.6 percent of the total variance. Lasher’s Anxiety of Aging Scale measures on a 5-point Likert scale, and items are scored from 1 (Strongly disagree) to 5 (Strongly agree). A higher score indicates a higher level of aging anxiety [15].

### 2.5. Statistical Analysis

The Lasher Anxiety of Aging Scale’s reliability was evaluated using internal consistency and item-to-total correlation [corrected for overlap]. Cronbach’s α values of ≥0.7 and item-to-total correlation of <0.4 are considered acceptable. The construct validity of the AAS was assessed by performing confirmatory factor analysis (CFA) and the Rasch model. A diagonally-weighted least squares (DWLS) estimator was used as an appropriate estimator for the CFA’s ordinal data (i.e., Likert-type scale). Further, to assess the model fit, the following fit indexes were considered: Comparative Fit Index (CFI), Tucker-Lewis Index (TLI), Root Mean Square Error of Approximation (RMSEA), Standardized Root Mean Square Residual (SRMR) and Chi-square (χ2) test. The acceptable model fit was defined as CFI and TLI > 0.9, RMSEA and SRMR < 0.08, and a non-significant Chi-square.

Average Variance Extracted (AVE) and Composite Reliability (CR) were computed using the factor loadings extracted from the CFA. Values > 0.5 and >0.7 for AVE and CR indicate evidence of the convergent validity of the AAS. To further assess the AAS, Rasch analyses were performed using the partial credit model [15,16,17,18]. Item fit in the Rasch model was evaluated using information-weighted fit statistic (infit) mean square (MnSq) and outlier-sensitive fit statistic (outfit) MnSq. Values between 0.5 and 1.5 were considered as an acceptable fit. Additionally, the person and item separation indices and their reliabilities were computed for each AAS subscale. Permissible values for person and item separation reliability were >0.7, while that for the person and item separation indices were >2. To evaluate measurement invariance across different subsamples of older adults with gender and living status, the differential item functioning (DIF) was carried out in each item of the AAS. A DIF contrast < 0.5 logits indicates a slight absence of DIF. All statistics were performed using IBM SPSS Statistics version 26.

## 3. Results

The results of CFA showed that the four-factor structure model fits well with the data (χ2 = 593.844, df = 164, *p* < 0.001; CFI = 0.933; TLI = 0.907; RMSEA = 0.076 (90% CI 0.069–0.084), *p* < 0.000), SRMR = 0.070). All factor loadings were significant and ranged from 0.54 to 0.85 (item 16) to 0.85 (item 2).

The corrected item-total correlations exceeded the recommended threshold of a value of 0.40, ranging from 0.40–0.80 (Table 2).

Item-fit statistics are shown in Table 2. The Infit and Outfit MNSQ lay within the acceptable range of 0.5–1.5. For the older adults, the most challenging item was ‘I have never lied about my age in order to appear younger’, whereas the most comfortable item was ‘I will have plenty to occupy my time when I am old.’ Additionally, all items were consistent across gender and living status categories.

The subscale level analysis of the items yielded item separation indices and person separation indices. The item separation indices ranged between 3.41 and 10.42, with a reliability range between 0.87 and 0.99. The person separation indices ranged between 2.30 and 2.86 while the person reliability ranged between 0.73 and 0.81 (Table 3).

Besides the previous results, an independent samples *t*-test was performed to examine the difference between aging anxiety among men and women. One-way ANOVA was also used to assess the difference between marital status and educational level with the anxiety of aging. The results showed that there was no significant correlation between gender (*p*-value = 0.311), educational level (*p*-value = 0.52), and marital status (*p*-value = 0.86) with a mean score of anxiety of aging.

As Table 3 shows, all the Cronbach’s alpha, CR, and AVE values were above the acceptable thresholds in four subscales.

## 4. Discussion

In the present study, the Persian version of the AAS’s psychometric properties was examined using classical test theory (CTT) and item response theory (IRT) approaches. CTT is based on the fact that a person’s score is a set of true scores and error scores. However, CTT does not clearly picture the relationship between items and attributes questions to the subject’s ability to measure them [19]. In this CTT, for all abilities (subjects with different abilities), a constant value of measurement standard error is obtained. Hence, the reliability index remains constant for all ability levels. Of course, the accuracy of measuring each test varies at different levels of ability. This problem is not evident in the case of CTT. Unlike CTT, in IRT, the index of accuracy of parameter estimation is called the information function, and its error rate is equal to the standard error of parameter estimation. The results of the CTT indicated that the Persian version of the AAS was internally consistent and had an acceptable four-factor structure using CFA and demonstrated acceptable AVE and CR across older adults. In addition to this, the Rasch model further confirmed that the Persian version of the AAS had a four-factor structure and was consistent across gender and living status groups. Previous studies also supported the four-factor structure of the AAS [15,16,17,18]. However, our study was the first study to apply it, particularly to older adults.

Generally, in the context of cross-sectional studies, DIF can be defined as the different difficulty of the AAS items across group membership [19]. Another important contribution of our results is to confirm the measurement invariance of the AAS across gender and living status among older adults in Iran. Therefore, both males and females, as well as those who were living lonely and with family, perceived all items in a similar pattern.

The participants in this study had some difficulties with understanding item 4 of the AAS (physical appearance: “I have never lied about my age in order to appear younger”). One potential reason for this is that our older adults had younger ages than other studies (69.4 years). Therefore, it might be that few participants had tried to falsely present themselves as young. The current study results showed no statistically significant difference between the mean score of anxiety of aging and gender, educational status, and marital status. According to this study, societal values and traditions have a greater impact on aging concern than demographic variables.

A fundamental principle in examining the measurement invariance is to compare the individuals from diverse subgroups to detect bias at the item or scale level. The study results revealed that the AAS items were consistent across gender and living status among the participants’ subgroups. Consistent with previous studies [17,18], the AAS can be conveniently applied for all gender groups and living conditions of older adults in Iran.

### 4.1. Limitations

As with any study, our study also has some limitations. Firstly, as older adults with Kurdish and Turkish cultures have participated in this study and since aging anxiety can be influenced by culture, we suggest that the readers be cautious in the generalization of the results. Secondly, most of the participants in the study are illiterate, and therefore, completing the questionnaire through the face-to-face method took a long time.

### 4.2. Strengths of the Study

Despite the limitations mentioned above, using a large sample size can be considered as one of the strengths of the present study.

### 4.3. Recommendations

Since it is necessary to perform population-based interventions to reduce aging anxiety, it is suggested that future studies examine the factors affecting the anxiety of aging in Iranian older people. Although no significant relationship was observed in this study between demographic variables and anxiety in old age, it is recommended that researchers re-examine these variables in future studies because, in different cultures, different factors can affect anxiety of aging.

## 5. Conclusions

In general, the AAS has robust psychometric properties to measure anxiety levels among the older adult population of Iran. The questionnaire’s validity and reliability and the factor structure of the items are consistent and acceptable. Therefore, the present study results can be applied in diverse circumstances, including mental health research of the older adult, with high performance.

## Figures and Tables

**Table 1:** Sociodemographic characteristics of the study participants (*n* = 703).

Variable	Mean ± SD or *n* (%)
Age (Year)	69.41 ± 8.11
Gender	
Male	416 (59.2%)
Female	287 (40.8%)
Educational status	
Illiterate	560 (79.7%)
Primary and secondary schools	143 (20.3%)
Living status	
With wife/husband	468 (66.6%)
With children	114 (16.2%)
Alone	121 (17.2%)
Smoking and Alcohol	
Current smoker (Yes)	118 (16.8%)
Alcohol drinker (Yes)	12 (1.7%)
Occupational status	
Retired	138 (19.6%)
Employed	105 (24.6%)
Unemployed	39 (15.8%)
Housekeeper	238 (40.0%)

**Table 2:** Psychometric properties of the Anxiety about Aging Scale on the item level.

Item # and Question	Analyses from Classical Test Theory		Analyses from Rasch				
	Factor Loading ^a^	Item-Total Correlation	Infit MnSq	Outfit MnSq	Difficulty	DIF Contrast Across Gender ^b,c^	DIF Contrast Across Living Status ^b,d^
1. Enjoy being around old people	0.66	0.73	1.39	1.01	0.31	−0.13	−0.18
3. Like to visit my older relatives	0.66	0.68	1.29	1.22	0.40	−0.10	0.19
10.Enjoy talking with old people	0.81	0.80	0.69	0.70	0.13	−0.26	0.31
13. Feel very comfortable around an old person.	0.80	0.72	0.97	1.11	−0.59	0.22	−0.05
19. Enjoy doing things for old people.	0.77	0.68	1.0	0.85	−0.25	0.12	−0.37
5. I fear it will be very hard for me to find contentment in old age.	0.83	0.40	1.21	1.03	0.19	−0.49	−0.45
7. I will have plenty to occupy my time when I am old.	0.64	0.43	1.11	1.19	−1.59	0.08	0.22
11. I expect to feel good about life when I am old.	0.59	0.55	0.81	0.53	0.60	0.10	−0.30
16. I believe that I will still be able to do most things for myself when I am old.	0.54	0.41	1.39	1.24	0.39	0.40	−0.04
18. Expect to feel good about myself when I am old.	0.67	0.59	0.72	0.59	0.42	−0.07	−0.18
4. Have never lied about my age in order to appear younger.	0.83	0.50	1.39	1.11	1.01	−0.25	−0.28
9. It doesn’t bother me at all to imagine myself as being old.	0.61	0.47	1.36	1.24	−0.35	−0.25	0.39
12. Have never dreaded the day I would look in the mirror and see gray hairs.	0.80	0.69	0.72	0.61	0.02	0.27	0.0
15. Have never dreaded looking old.	0.84	0.68	0.75	0.66	−0.05	0.25	−0.28
20. When I look in the mirror, it bothers me to see how my looks have changed with age.	0.55	0.46	1.26	1.34	−0.64	−0.06	0.0
2. Fear that when I am old, all my friends will be gone.	0.85	0.70	0.76	0.76	0.26	−0.08	−0.06
6. The older I become, the more I worry about my health.	0.81	0.71	0.85	0.52	0.65	−0.16	0.13
8. Get nervous when I think about someone else making decisions for me when I am old.	0.62	0.43	1.24	1.38	−0.95	−0.05	0.30
14. Worry that people will ignore me when I am old.	0.85	0.69	0.78	0.68	0.12	0.14	−0.13
17. Afraid that there will be no meaning in life when I am old.	0.82	0.70	0.64	0.67	−0.09	0.09	−0.37

^a^ Based on confirmatory factor analysis. ^b^ DIF contrast > 0.5 indicates substantial DIF. ^c^ DIF contrast across gender = difficulty for females-difficulty for males. ^d^ DIF contrast across living status = difficulty for participants who were living alone-difficulty for participants who were living with family. MnSq = mean square error; DIF = differential item functioning.

**Table 3:** Psychometric properties of the Anxiety About Aging Scale at the scale level.

Psychometric Testing	Fear of Old People	Psychological Concerns	Physical Appearance	Fear of Losses
Composite Reliability	0.90	0.79	0.85	0.89
Average Variance Extracted	0.55	0.43	0.54	0.63
Internal consistency (Cronbach’s α)	0.88	0.70	0.74	0.76
Internal consistency of the original English language version	0.78	0.74	0.74	0.69
Person separation index	2.30	2.81	2.86	2.79
Item separation index	3.41	10.42	5.13	8.52
Person separation reliability	0.73	0.81	0.79	0.79
Item separation reliability	0.87	0.99	0.96	0.99

## Data Availability

The data presented in this study are available on request from the corresponding author (H.A).

## Appendix A. Persian Version of Lasher and Faulkender Anxiety about Aging Scale

.من از بودن کنار افراد سالمند لذت می برم	1
.می ترسم وقتی که به سن سالمندی رسیدم همه دوستانم را از دست بدهم	2
.دوست دارم اقوام و آشنایان سالخورده را ملاقات کنم	3
.هیچ وقت برای اینکه جوانتر به نظر برسم راجع به سن و سالم دروغ نمی گویم	4
.ازاینکه در دوران سالمندی به سختی بتوانم احساس شادمانی داشته باشم، نگران هستم	5
.هرچه که سن من بالاتر می رود، بیشتر نسبت به سلامتی خود نگران می شوم	6
.هنگامی که به سن سالمندی برسم فرصت زیادی برای مشغول کردن خود خواهم داشت	7
.از اینکه در دوران سالمندی کسی بخواهد برایم تصمیم گیری کند، عصبی می شوم	8
.از اینکه خودم را پیر تصور کنم، ناراحت نمی شوم	9
.از مصاحبت و همنشینی با افراد سالخورده لذت می برم	10
.انتظاردارم که هنگام پیری، حس خوبی از زندگی خواهم داشت	11
.هرگز از دیدن موهای سفید خود در مقابل آینه، ناراحت نمی شوم	12
.وقتی با یک فرد پیر هستم احساس خیلی خوبی دارم	13
.نگران هستم که وقتی پیر شدم بقیه من را کنار بگذارند	14
.هرگز از اینکه پیر دیده می شوم، ناراحت نمی شوم	15
.باور دارم که هنوز می توانم بیشترکارها را، خودم انجام دهم	16
.نگران هستم که در زمان پیری، زندگی پوچ و بی هدف خواهد بود	17
.انتظار دارم که در زمان پیری، حس خوبی در مورد خودم داشته باشم	18
.از اینکه کاری برای افراد سالخورده انجام دهم لذت می برم	19
.نگریستن به تغییر قیافه و دیدن قیافه سالخورده خود در آینه آزارم می دهد	20

## Appendix B. English Version of Lasher and Faulkender Anxiety about Aging Scale

1.	I enjoy being around old people.
2.	I fear that when I am old all my friends will be gone.
3.	I like to go visit my older relatives.
4.	I have never lied about my age in order to appear younger.
5.	I fear it will be very hard for me to find contentment in old age.
6.	The older I become, the more I worry about my health.
7.	I will have plenty to occupy my time when I am old.
8.	I get nervous when I think about someone else making decisions for me when I am old.
9.	It doesn’t bother me at all to imagine myself as being old.
10.	I enjoy talking with old people
11.	I expect to feel good about life when I am old.
12.	I have never dreaded the day I would look in the mirror and see gray hairs.
13.	I feel very comfortable when I am around an old person.
14.	I worry that people will ignore me when I am old.
15.	I have never dreaded looking old.
16.	I believe that I will still be able to do most things for myself when I am old.
17.	I am afraid that there will be no meaning in life when I am old.
18.	I expect to feel good about myself when I am old.
19.	I enjoy doing things for old people.
20.	When I look in the mirror, it bothers me to see how my looks have changed with age.
